# A Qualitative Assessment of the Evidence Utilization for Health Policy-Making on the Basis of SUPPORT Tools in a Developing Country

**DOI:** 10.15171/ijhpm.2016.158

**Published:** 2017-01-08

**Authors:** Mohammad Hasan Imani-Nasab, Hesam Seyedin, Bahareh Yazdizadeh, Reza Majdzadeh

**Affiliations:** ^1^Social Determinants of Health Research Center, Lorestan University of Medical Sciences, Khorramabad, Iran.; ^2^Department of Health Services Management, School of Medical Management and Information Sciences, Iran University of Medical Sciences, Tehran, Iran.; ^3^Knowledge Utilization Research Center, Tehran University of Medical Sciences, Tehran, Iran.; ^4^Department of Epidemiology & Biostatistics, School of Public Health, Tehran University of Medical Sciences, Tehran, Iran.

**Keywords:** Health Policy-Making, Evidence-Based Health Policy-Making, Utilization of Evidence, Iran

## Abstract

**Background:** SUPPORT tools consist of 18 articles addressing the health policy-makers so that they can learn how to make evidence-informed health policies. These tools have been particularly recommended for developing countries. The present study tries to explain the process of evidence utilization for developing policy documents in the Iranian Ministry of Health and Medical Education (MoHME) and to compare the findings with those of SUPPORT tools.

**Methods:** A qualitative research was conducted, using the framework analysis approach. Participants consisted of senior managers and technicians in MoHME. Purposeful sampling was done, with a maximum variety, for the selection of research participants: individuals having at least 5 years of experience in preparing evidence-based policy documents. Face-to-face interviews were conducted for data collection. As a guideline for the interviews, ‘the Utilization of Evidence in Policy-Making Organizations’ procedure was used. The data were analyzed through the analysis of the framework method using MAXQDA 10 software.

**Results:** The participants acquired the research evidence in a topic-based form, and they were less likely to search on the basis of the evidence pyramid. To assess the quality of evidence, they did not use standard critical tools; to adapt the evidence and interventions with the local setting, they did not use the ideas and experiences of all stakeholders, and in preparing the evidence-based policy documents, they did not take into consideration the window of opportunity, did not refrain from using highly technical terms, did not write user-friendly summaries, and did not present alternative policy options. In order to develop health policies, however, they used the following innovations: attention to the financial burden of policy issues on the agenda, sensitivity analysis of the preferred policy option on the basis of technical, sociopolitical, and economic feasibility, advocacy from other scholars, using the multi-criteria decision-making models for the prioritization of policy options, implementation of policy based on the degree of readiness of policy-implementing units, and the classification of policy documents on the basis of different conditions of policy-making (urgent, short-term, and long-term).

**Conclusion:** Findings showed that the process of evidence utilization in IR-MoH enjoys some innovations for the support of health policy development. The present study provides IR-MoH with considerable opportunities for the improvement of evidence-informed health policy-making. Moreover, the SUPPORT process and tools are recommended to be used in developing countries.

## Background

A policy document is a decision-making tool which explains an urgent policy issue and offers and assesses different policy options.^1^ Evidence-informed policy-making can affect the policy-making process by means of the evidence searched systematically, appraised critically, and analyzed and synthesized precisely. Evidence is defined as the actual or asserted facts intended for use in support of a conclusion. A fact, in turn, is something known through experience or observation.^2^ Systematic reviews and meta-analyses are regarded as the best types of evidence to inform policies since they do not have the limitations of single studies.^3^ However, policy-making is not merely the presentation of the best policy options. Systematic reviews of effectiveness are necessary, but not sufficient for policy-making. In order to choose, or else, discard a policy option, policy-makers also need other information concerning the potential negative consequences of policy options such as their different effects on different subgroups of the society and their contributions to health inequality, the modifying factors in the effectiveness of policy options, the technical feasibility and acceptance of policy options among different stakeholders, and the costs and barriers of their implementation. Unless this evidence, in addition to the evidence related to the effectiveness of an intervention, is given to policy-makers, they will unlikely choose the best policy option.^4^ Global evidence must be interpreted in accordance with the policy-making setting; this requires the synthesis of the global and local evidence. Synthesized evidence such as a policy brief is superior to systematic reviews since it supports the decision-making in a specific context.^5^

There is an increasing international interest in encouraging health policy-makers and managers to follow the results of relevant high-quality research evidence.^2,6^ Although there are advanced methods of developing clinical guidelines and health technology assessments, there is little experience in the development of evidence-based policy documents.^7^ Some scholars hold that the main challenge facing the evidence-based policy-making is not to develop internationally valid evidence, but to develop systematic and precise methods for the identification, interpretation, and utilization of evidence in different policy-making contexts.^8^ How to utilize the health evidence in policy-making is a problem for both developed and developing countries.^9,10^ However, the low/middle-income countries have less resources to deal with their health system problems compared to rich countries, and in order to make the best use of their limited resources, they are in a more urgent need for high-quality evidence.^2^ A study in 2011 in the European Union (EU) showed that health policy-making units hardly had the necessary structures, procedures, and tools to make use of evidence-based policy-making.^11^ Findings of another study in the same year indicated that health policy-makers in Eastern Mediterranean countries did not generally engage in the process of evidence utilization.^12^

The organizations which support the governments in developing health policies and programs through the use of evidence rarely publish their guidelines for developing a policy document.^13^ Eighteen tools of evidence-informed health policy-making were introduced and explained in 2009 and published in the form of 18 articles through an international campaign called SUPPORT.^14^ Moreover, Department of Health in Victoria, Australia, published a guideline for evidence-informed policy and practice in 2010.^15^ Some recent studies have addressed the attitudes and perceptions of health policy-makers on the utilization of evidence in policy-making.^9,16,17^

In recent years, the utilization of research evidence in public policy-making, especially health policies, has received considerable attention in Iran. Among recent measures taken in Iran for the promotion of evidence-based decision-making, the following can be mentioned: the establishment of a research center by the National Parliament, the establishment of the Council of Health Policy in the Iranian Ministry of Health and Medical Education (MoHME), the establishment of the National Institute for Health Research and Health Technology Assessment Bureau in MoHME, the adoption of policy documents in the agenda of the Secretariat of the Health and Food Safety Council as the highest decision-making body in the government, and emphasis on the use of evidence in policy-making in the 2025 vision of Iran’s health system.^18-20^ The integration of health services and medical education has provided an invaluable opportunity to decrease the gap between knowledge and action.^21^ In spite of the developments and opportunities mentioned above, however, the process of utilization of evidence to develop policy documents has not been fully recognized in Iran.

Although more than 7 years have passed since the publication of the SUPPORT tools, one cannot easily find a study which deals specifically with the process of evidence utilization based on these tools. In the present study, the process of utilization of evidence to develop policy documents in MoHME is explained with a qualitative approach. The results can shed some light on the process of exploitation of evidence to produce health policy documents in a developing country. The present study compares this process with that recommended by SUPPORT. Furthermore, it has been claimed that the process and the tools proposed by SUPPORT can be used in every country regardless of their income.^2^ Therefore, the results give some clues about the feasibility of these tools in developing countries.

## Methods

### Setting and Study Participants

MoHME consists of 9 deputies, each deputy consisting of a few centers/bureaus, and each center/bureau consists of several technical offices. There are some technical offices in MoHME, for example, the Malaria Office. Such centers or bureaus as the Center of Communicable Diseases, in turn, consist of several relevant technical offices, and such under secretariats as the treatment consist of several other relevant centers or bureaus. Technical offices are usually the starting points for developing policy documents. The participants consisted of general managers of bureaus, heads of technical offices, and their senior technicians. A purposeful sampling was carried out to include a maximum variety of participants (25). To ensure utmost variety, the participants were chosen on the basis of their sex, field of study, academic degree, work experience, and employment status. The interviews were conducted with key informants who had at least 5 years of experience in preparing evidence-based policy documents.

### The Instrument

In order to direct the interviews towards the intended goal, the process of utilization of evidence, ‘the framework of utilization of evidence in policy-making organizations’ developed by Canadian Health Research Foundation (CHSRF), Ottawa, ON, Canada, was used. This is a simple and popular framework used for the explanation of evidence utilization in policy-making organizations. This framework has four stages including acquisition, assessment, adaptation, and application of evidence.^22^ The evidence consisted of all types of evidence such as research evidence, routine data, or knowledge and experience of different stakeholders.

The draft interview guide consisted of some general questions based on the CHSRF framework. The draft interview guide was piloted in 5 interviews, and based on the results, some improvements were made. The final questions were:

- How did you acquire the needed evidence to develop your policy document?
- Did you assess the evidence? If so, how?
- Did you adapt the proposed policy options to the local context? How?
- For whose information and how did you use the evidence?

### Data Collection

Face-to-face interviews with open-ended questions were carried out by the first author (MHIN), a PhD student in health policy with 12 years of experience in Iran’s Health System. There was no communication between the interviewer and the participants prior to the study. The interviews lasted approximately 32 to 78 minutes (53 minutes in average). Interviews continued until the stage of data saturation. Before conducting the interviews, the necessary information was provided on the goal of the study, and oral consent was obtained from every participant. Before conducting the study, the necessary permission was obtained from the Ethics Committee of Iran University of Medical Sciences, Tehran, Iran. Most interviews were carried out behind closed doors in every participant’s office and recorded by an electronic voice recorder.

### Data Analysis

All the recorded interviews were transcribed and coded on the basis of the framework analysis developed by Ritchie and Spencer. The framework analysis is suitable for summarizing and classifying the data within a conceptual framework. The framework analysis consists of 5 stages: familiarization, thematic identification, indexing, charting, and mapping and interpretation.^23^

After rereading the interview transcripts for several times and choosing the categories (the CHSRF framework), indexing was carried out on the basis of a previously prepared list. When new codes were found, they were added to the previous list. Eventually, the themes and subthemes were identified deductively and inductively (Table 1). The code-recode procedure was used for analyzing the data. Personal biases of the researchers could have affected the way the data were collected and interpreted. Although it is impossible to thoroughly eliminate the effect of personal characteristics of the researchers from the study, attempts were made to find and control these effects throughout the study and to decrease their impacts as much as possible. To ensure the trustworthiness of the findings, two members of the research team coded two interview transcripts, and the slight cases of mismatch were resolved. In order to receive feedback from the participants and make sure that their ideas and views were presented accurately, the findings were sent to them, and their feedback was used to improve the interpretation. MAXQD 10 software was used for coding and data management. Finally, a number of research methodologists examined, verified, and confirmed the design of the study. The COREG (consolidated criteria for reporting qualitative research) checklist was used for reporting this study.

**Table 1:** Characteristics of the Participants in the Explanation of Utilization Process of Evidence in Health Policies in Iran

**Characteristics**	**Gender**		**Experience (y)**			**Background**		**Employment Status**			**Academic Degree**			**Management exp.**	
	F	M	5-10	11-20	21-30	Clinical	Non-clinical	Senior technician	Head of technical office	General managers of bureau	PhD	GP	MS	Has	Does not have
**#**	7	18	5	17	3	11	14	8	10	7	5	3	17	17	8

## Results

The findings are based on the four categories of acquisition, assessment, adaptation, and application (Table 2). Samples of direct quotations from the participants have been presented inside quotation marks in italics. Table 1 shows the information of 25 study participants. None of the invitees refused to interview.

**Table 2:** The Coding System

**Category**	**Theme**	**Subtheme**
Acquisition	Definition of policy issue	-
	Thematic search	The effects of the policy options
		The feasibility of the policy options
	Methodological Search	Systematic reviews
		Cost-effectiveness studies
		Qualitative studies
	Global evidence	-
	Local evidence	Domestic studies
		Routine data
		Ideas and experiences of stakeholders
Assessment	Quality assessment of the evidence	Quality assessment
		Local applicability
		Effects on equity
	Ideas and experiences of stakeholders	Fair representation of all stakeholders
		Abiding by the Chatham House Rule
		The skill of the facilitator
Adaptation	Global evidence	-
	Local evidence	Domestic studies
		Pilot implementation
		Ideas and experiences of stakeholders
		Routine data
	Synthesis of evidence	-
Application	User-friendly report	Graded-entry format
		Brevity‏
		Not using technical language
		Considering the window of opportunity
	Advocacy	-
	The report structure	Graded-entry format
		Matrix of policy options
		Sensitivity analysis
		Multi-criteria‏ decision**-**making‏ model
	Reporting	Written
		Oral

### Acquisition of Evidence

Acquisition of evidence refers to the definition of a specific policy issue, searching, and acquiring the appropriate evidence about it and/or ordering the evidence from a third party.^24^ In order to develop health policies, the participants search for two types of evidence: the effects of policy options, and their feasibility. Almost, all interviewees search for the effects of policy options in the scientific databases of the Internet. In order to search for the evidence related to feasibility, however, they search for the routine data and ideas and experiences of stakeholders.

The evidence is usually searched in a thematic form. The interviewees usually search for the evidence related to the positive effects of the policy options rather than their negative effects. Some of the interviewees also pay special attention to the cost-effectiveness of the interventions:

*“When I want to give the task of searching for the evidence to a technician, I surely ask him for studies of cost-effectiveness. The reason is that we are investors here, and if the intervention is not cost-effective, we will not carry it out at all; we want to spend the saving money on other problems. *”

Most interviewees stated that they faced a number of problems while searching for evidence.

*“In the searching for the basic insurance package, we couldn’t find any systematic reviews or economic evaluations; most of the studies were either descriptive or qualitative. *”

Rather than searching for original evidence, some of the interviewees preferred to use synthesized evidence from reputable organizations such as World Health Organization (WHO) or the Center for Disease Control (CDC) in America. They believed that such evidence is synthesized, and therefore, it is valid since its methodological quality has been already assessed and it speeds up the process of preparing policy documents.

The interviewees usually searched for the evidence of economic and technical feasibility of policy options; they were less likely to search for the evidence related to social/political feasibility. Most participants did not believe that feasibility studies conducted in other countries are of any help to them. They also stated that domestic evidence of feasibility is rarely published, so in most cases they collected and analyzed raw data.* “We search for studies of feasibility of intervention; if there is a study, we will use it, and if not, by holding meetings with stakeholders, we will try to find out how much the intervention is economical, and how it is socio-politically, and technically feasible.”*

When faced with a lack of evidence for assessing feasibility of policy options, some of the interviewees turned their attentions to pilot implementation of policy option which has a higher chance of being accepted by the policy-making authorities. Moreover, some of the interviewees stated that when there is no domestic evidence for the effectiveness of programs, some policy-makers prefer to find it in research institutions.

*“In the phenylketonuria screening program, the evidence from other countries showed the effectiveness of intervention. However, the conditions of those countries were so different from ours; we were not convinced the evidence works in our country; we deemed it more appropriate to pilot the intervention and measure its effects on our own without help from others. *”

A small number of the interviewees actively searched for the qualitative studies in order to assess the feasibility of policy options. One of the interviewees believed that if, in developing a policy, no attention is given to the qualitative evidence, the policy will surly fail. Other participants believed that the qualitative studies in other counties would not help policy-making in their country.

*“Qualitative evidence is only usable in the setting in which the study has been carried out, therefore, our search does not include foreign qualitative evidence. *”

Some of the interviewees stated that the type of their search for evidence could be different depending on the amount of time they had for preparing a given policy. If they have enough time to search for original evidence, they prefer to search, assess the quality, and synthesize the evidence themselves. However, if they do not have enough time, they will use the knowledge and experience of other local researchers who have already published some articles about the intended policy issue. If they have a limitation of time, they will use the tacit knowledge of available researchers usually in Tehran to offer policy options.

### Assessment of Evidence

Assessment of evidence refers to the assessment of the quality of evidence, its effects on equity, and its applicability in the policy-making setting.^24^ The findings related to the assessment of evidence are presented regarding the differentiation of research evidence, routine data, and the ideas of stakeholders. None of the participants used the standard tools of critical appraisal such as CASP and GRADE to assess the methodological quality of the research evidence.^25,26^ Some of the participants believed that they were good tools for assessing the methodological quality of the research evidence and useful for evidence producers rather than for evidence users. They stated that they needed a tool that would help them accept or reject an article. A number of the interviewees believed that some aspects of quality of research studies could not be assessed on the basis of the texts, and therefore, they preferred to commission research and directly control the production of studies.

*“You cannot assess some of the points from the text; they must be controlled while conducting the study. Hence, we mostly prefer to order research ourselves and have supervision over the process rather than use the results of studies we aren’t aware of their quality control.*”

Most of the participants used other simple ways for evidence assessment. Some assessed the quality of the articles with their own knowledge of research methodology. Some others judged the quality of the evidence on the basis of the reputation of the journals in which it was published, its impact factors, or its publication in specialized journals. Some participants assessed the quality of original articles in terms of the evidence pyramid and tried to use the upper-level evidence to develop the policy documents. The criterion for some participants was the international reputation of the organization funding the study or that of the organization which used the results of the study.

*“A yardstick of quality of evidence is the organization which pays the budget for the project. Another clue is the use of evidence by reputable organizations such as WHO, Harvard, and World Bank in their own plans and policies.*”

Almost all interviewees were in doubt about the quality of the routine data. Some held that the lack of quality control over these data was the reason for their mistrust. The quality of the evidence obtained from the stakeholders in face-to-face meetings depends on following the principles of policy dialogues, such as making sure that the stakeholders in the meeting are the representative sample of all potential stakeholders, abiding by the Chatham House principle (ie, not revealing the identity of the participants of the dialogue) and using an impartial and skillful facilitator. Given that only available individuals are invited to the policy dialogue and there is no sufficient knowledge of the principles of policy dialogue, the evidence does not usually enjoy the expected quality.

Only a small number of participants considered the effects of policy options on equity and proposed strategies to eliminate or decrease these effects in their policy documents.

*“To select the appropriate type of food for iodine addition, we considered the accessibility of food to all people. We chose salt for our purpose; both the poor and rich add salt to their food every day, and if iodine is added to salt, its price won’t increase very much.*”

A small number of the interviewees believed that as long as adaptation is one of the stages of evidence utilization for developing a policy, it is not necessary to assess the local applicability of policy options.

*“For example, Franchise is used in developed countries to decrease the moral hazards; in my opinion, it is not scientifically valid to check if the intervention is compatible with my country or not; we adapt the preferred policy option eventually. After all, we adjust the intervention.*”

Nevertheless, some of the interviewees stated that they assessed policy options on the basis of the similarities of the conditions under which they have been implemented. In other words, they tried to propose policy options which had been successful under similar political, economic, and social conditions. Some other participants pointed to the requirements of the health systems of successful countries, and they compared their conditions with our health system. They believed that the more their requirements are similar to ours, the more the policy is effective, and the less we need contextualization.

*“A policy which works in Afghanistan will probably work here, and there isn’t much need for adaptation. However, it is not clear whether a successful policy in Canada will have the same results here too; it may not work here and it may need more adaptation, both in terms of design and implementation.*”

### Adaptation

Adaptation refers to the synthesis of various types of evidence (global and local) and adapting policy options to the local conditions if necessary.^24^ The majority of the interviewees believed that the adaptation of policy options to the local conditions is the most important and complicated step in the evidence utilization for policy-making. A number of participants stated that since there are no standardized methods for the adaptation of policy options, the output of the processes will be different depending on the type of the evidence used. Some of the participants stated that the reason for the inefficiency of most imported policies is their inappropriate adaptation.

*“Sometimes, we borrow the policy intact without any change, and sometimes, we change it so much that we can’t say if it’s the same policy or not, and therefore, it may not be effective.*”

The participants used three types of evidence to adapt the policy options: The ideas and experiences of the stakeholders, the published domestic evidence, and the results of pilot implementations. Only a small number of participants tried to use the ideas of all the stakeholding groups such as the receivers and providers of health services. Most technical bureaus of MoHME organized the technical/scientific committees consisting of salient scholars and executives who lived in Tehran and used their help in the adaptation of policies.

*“Hospital accreditation has two models; functional and departmental. In various meetings with executive elites, we concluded that in view of our organizational culture, we should design the hospital accreditation on the basis of functions but implement it on the basis of departments. This has never been experienced nor recommended in any of the international endeavors.*”

### Application of Evidence

Application of evidence is concerned with the structure and user-friendliness of policy documents (being brief, having graded entry format, not using technical language, and taking into account the window of opportunity) to inform the different stakeholders.^24^ The interviewees used the evidence-based policy documents to inform policy-makers, policy-making authorities, and key stakeholders. Most of the participants referred to advocacy as one of the functions of evidence-based policy documents. A small number of interviewees paid attention to brevity, refraining from technical language, and the window of opportunity.

*“I try to prepare a summary so that a policy-maker will read it without getting bored; I do not use technical language; I use the policy-maker’s language. I also consider the window of opportunity when I submit the document.*”

Although the participants prepared the policy documents in written forms and sent them to policy-makers, they often presented the documents to the stakeholders in the form of lectures using the PowerPoint software. The participants used different frameworks for the preparation and presentation of the documents. Most of them used a framework introduced by the Health Policy Secretariat of MoHME named “the Policy Document.” In this document, a policy issue is explained and the best option is introduced to policy-makers. A small number of participants used other frameworks such us 1-3-25, Executive Summary, Policy Brief, and the Framework for Strategic Program Report.

*“Based on the framework presented by the Council of Policy on the website, we prepared the document. Our document introduced an analysis of the existing conditions; some recommended interventions including their strengths and weaknesses and some guidelines for implementation.*”

Although most of the interviewees reviewed the existing policy options in their proposed documents, they introduced one final policy option to the policy-makers and the stakeholders as their desirable option and tried to attract their attentions towards it by means of different evidences.

*“We presented our final policy document in the Council of Deputies, in the presence of the minister himself. We stated that the ‘Accreditation Model’ is the only model which has originated in the health sector and the rest of the models come from industry and their literature is very industrial.*”

However, some of the interviewees were against the introduction of a single preferred option by the policy-developing team. They believed that the policy-makers decide what option is the best in terms of feasibility.

Most of the participants pointed to the positive effects and the economic feasibility of the proposed policy options in the policy documents although a small number of them put emphasis on the negative effects, cost-effectiveness, social/political, and technical feasibility of the policy options.

*“In our presentation, we reported the effectiveness of the intervention and elaborated on the implementation steps, the possible executive barriers, the needed financial resources, and the results of our meetings with stakeholders.*”

One of the interviewees analyzed the sensitivity of a preferred policy option in terms of three potential feasibility scenarios, believing that this analysis will provide the policy-makers with the prerequisite knowledge of different consequences of the preferred policy option if financial, technical, and political support decreased.

*“For the most cost-effective intervention at the national level, an evaluation of capacities is conducted and submitted to the policy-makers in three scenarios. One scenario is the ideal condition in which all the capacities including human, financial, and other resources are complete. In this scenario, the whole project will be finished in two years, for example. In another scenario, 70% of the human resources, 80% of the financial resources, and 50% of other resources are complete, and it takes three years. We give these in different scenarios to the policy-maker to make the decision-making easier.*”

Another interviewee, after preparing and finalizing his policy document, held a meeting with a large number of scholars and put his document under their critical scrutiny. By so doing, he not only put his policy document under test, but he also gained their support, and in this way, he drew the attentions of the policy-making authorities to his prepared policy document. Finally, he prioritized his policy options by means of multi-criteria decision-making models.

*“I use the ideas of 50 to 100 scholars to criticize my work; if we manage to gain the support of 60-70 scholars, we will have a good chance of having our policy option ratified and implemented.*”

Table 3 presents a comparison between the process of evidence utilization for health policy-making in MoHME and that recommended by the SUPPORT tools package.

**Table 3:** A Comparison Between the Process of Evidence Utilization for Health Policy-Making in MoHME and That of the SUPPORT Tools Package

	**SUPPORT Tools Package**	**MoHME Process**
Acquisition	- Search of evidence on the basis of types of evidence (evidence pyramid, qualitative, quantitative, etc) - Search of evidence on the basis of positive and negative effects, cost/cost-effectiveness, barriers of implementation, and acceptability of policy issues/options	- Thematic search of evidence rather than types of evidence - Search of positive effects rather than holistic search - Inaccessibility to optimal evidence and the use of brainstorming or asking for research studies - Use of pilot implementation rather than search of feasibility studies
Assessment	- Use of critical appraisal tools for assessing the quality of research evidence - Assessment of policy options’ effects on the equity - Assessment of local applicability of the research findings in the policy-making setting	- Not use of critical appraisal tools for assessing the quality of research evidence - Non-assessment of the effects of policy options on equity - Assessment of local applicability of the research findings merely on the basis of similarity in political, economic, and social conditions
Adaptation	- Organizing the policy dialogues with a representative sample of all the potential stakeholders - Using the results of process evaluation studies to identify the primary and secondary components of the policy options	- Using the local evidence, the results of pilot implementations, the views and experiences of scholars and managers, not every stakeholder - Non-using the results of process evaluation studies
Application	- Using the policy brief matrix with at least three policy options - Assessing the policy options on the basis of these criteria: positive and negative effects, cost/cost-effectiveness, barriers of implementation, and acceptability of policy issues/options	- Proposing only one preferred policy option - Sensitivity-analyzing the preferred policy option on the basis of potential scenarios - Putting the developed policy under critical scrutiny of other scholars - Using the multi-criteria decision-making models for prioritization of policy options - Using the patterns of development of evidence-based policy documents compatible with emergency, short-term, and long-term conditions

Abbreviation: MoHME, Ministry of Health and Medical Education.

## Discussion

The present qualitative study was conducted to explain the process of evidence utilization for the development of policy documents in MoHME. The findings were compared via SUPPORT tools package and with those of a guide for evidence-informed policy and practice (GEPP), which is among the gray literature.

In MoHME, the searches are often carried out on a thematic basis rather than on the basis of evidence pyramid. In order to support the development of health policies, there is an emphasis on the evidence related to the positive effects of the policy options and their economic feasibility. The evidence related to the negative effects and technical/political feasibility of policy options is rarely searched and used. In the SUPPORT package, it is recommended to search the evidence on the basis of both positive and negative effects of policy options, their cost-effectiveness, acceptability, and implementation barriers.^27^ GEPP also recommends the use of both positive and negative consequences of an intervention. This guide emphasizes that the searches for evidence should start with systematic reviews and cost-effectiveness analyses and continues with qualitative studies.^15^

In most cases, when participants face a problem accessing optimal evidence, they use techniques such as brainstorming or ordering research studies. In GEPP, in case of inaccessibility of quantitative evidence on the effectiveness, utilization of the program theory, program logic, or experts’ panel is recommended.^15^ One of the tools of the SUPPORT package gives some recommendations to deal with “the no-evidence” conditions.^28^ Most participants in the study conducted by Ellen et al also believed that the available research evidences are not usually relevant to the policy issues.^16^ Some of the participants in the study of El-Jardali et al stated that the available research evidences do not provide sufficient information on the effects and costs of policy options.^9^ Moreover, most participants in the study of Hyder et al pointed out that the researchers in developing countries are not capable enough to conduct health-policy-related research.^17^

One of the strategies of the participants to measure the feasibility of the policy options was piloting the policy option which had a higher chance of being approved by the policy-makers. Pilot implementation in smaller scales has also been recommended in GEPP in cases of lack of sufficient evidence.^15^

Although the participants of the study believed that the qualitative studies conducted in other countries are not of much help to policy-making in Iran, it has been emphasized in SUPPORT that the evidence related to barriers of implementation in other contexts and the lessons learned may be beneficial, though not sufficient.^29^

The interviewees did not use the standard critical appraisal tools for assessing the methodological quality of the evidence; instead, they used other ways such as their owns knowledge of research methodology, attention to impact factors of the journals, or whether the evidence is published in specialized journals, attention to the evidence pyramid, and attention to the organization which has funded the research or used the evidence for the development of its own plans and policies. In GEPP, the assessment of research evidence based on the evidence pyramid has been emphasized, and a table for assessing the qualitative and quantitative evidences has been provided.^15^ The participants of the study conducted by Albert et al preferred the studies which had been commissioned. They believed that methodology and references were an index for the quality of evidence, and they were more likely to trust the studies published in specialized journals. They also stated that they had more confidence in the research projects funded by reputable international organizations.^30^ Although the criteria used by the participants can give some clues about the quality of the research evidence, the standard critical appraisal tools are recommended. The evaluation of the quality of articles by referees and the fact that they exist in the scientific databases can increase the validity of the assessment and can speed up of the production process of evidence-based policy documents.

Considering the fact that only available persons are invited to policy dialogues and there is no sufficient knowledge of other principles of policy dialogues, the evidence gained from these dialogues does not have the required quality. One of the tools in SUPPORT deals with the way policy dialogues should be held. The recommendations provided by this tool can increase the quality of the evidence gained from the policy dialogues.^31^

The participants rarely consider the effects of policy options on the equity, and they rarely suggest some strategies to decrease or eliminate these effects in their policy documents. One of the tools in the SUPPORT package specifically assesses the effects of policy options on the equity. In GEPP, moreover, a table has been provided for the classification of inequalities in the health system.^15^

Some interviewees tried to propose policy options reported to be successful under similar political, economic, and social circumstances in the country. The participants in the study of Ellen et al stated that although there may be relevant research from other countries, they cannot be easily adapted to a different policy-making context. They also believed that a specific research is better than general one.^16^ The participants of the study of El-Jardali et al study emphasized the necessity of training the policy-makers on the methods of acquisition, assessment, and adaptation of policy options.^9^ One of the tools in SUPPORT deals with the assessment of local applicability of the systematic review findings in different settings and poses various questions about the possibility of their use in the local context such as the similarities of the findings in different contexts, the effect of on-the-ground realities and constraints on the feasibility of interventions, the effect of different arrangements of health system on the effectiveness of interventions, and the effect of baseline conditions on the absolute effects of the interventions. Similarly, in GEPP, this issue has been addressed under the ‘assessment of generalizability’ and has emphasized that judgments on generalizability must take place on the basis of the requirements of the policy-making context.^15^

In order to adapt policy options to the local context, the participants made use of the views and experiences of scholars, managers, local evidence, and the results of the pilot implementation. SUPPORT emphasizes that in order to adapt the policy options, the ideas of all stakeholders should be considered, but piloting has not been recommended as a method for adapting the policy options.^32,33^ Although the identification of primary and secondary components of an intervention has been considered by SUPPORT as essential for the adaptation of the policy options (process evaluation), most participants do not pay attention to this important consideration.^34^

The participants in the studies of Ellen et al and El-Jardali et al pointed to the necessity of brevity and refraining from technical language at the time of developing a policy document. The participants in the study of El-Jardali et al also stated that the majority of studies do not contain actionable messages.^9,16^

In SUPPORT, it has been recommended that at least three policy options should be provided in the policy brief. However, the participants usually introduce only one option as their preferred option to the policy-makers and stakeholders.^27^ The least expectation from a policy brief is that it should be able to compare the presence and the absence of the best policy option.

Neither the SUPPORT package nor GEPP deals with such innovations as the use of sensitivity analysis for the preferred policy option in terms of several feasibility scenarios, close examination of the proposed policy document by scholars, the use of multi-criteria decision-making techniques for the prioritization of policy options, and the presentation of a model for the development of evidence-based policy documents for long-term, short-term, and emergency conditions.

## Conclusion

Findings of this study revealed that the process of evidence utilization for the development of policies in MoHME does not fully confirm the process recommended by SUPPORT; it was also found that there are considerable opportunities for MoHME to improve the process. The present study was conducted in a qualitative manner, and therefore, its findings cannot be generalized to other contexts. Nevertheless, the findings give a clear picture of the process of evidence utilization for the development of health policies in a developing country. The comparison of the results with those of the SUPPORT Tool package and those of GEPP provided an opportunity to challenge the process recommended by these tools. One research recommendation is to assess the usability of SUPPORT tools for evidence-informed policy-making in other developing countries.

Even though the process of developing the evidence-based policy documents in IR-MoH does not match the process recommended by WHO (SUPPORT), the participants had innovations in this area, which are valuable and deserve careful attention. The following themes have not been addressed in the relevant literature: attention to financial burden of policy issues as the most important evidence on a policy agenda, sensitivity analysis of the preferred policy option in terms of various technical, social/political, and economic feasibility, advocacy from other scholars by examining the proposed policy option, the use of multi-criteria decision-making models for the prioritization of policy options, the implementation of policies on the basis of readiness of the policy-implementing units, and the classification of policy-document procedures on the basis of different policy-making conditions (urgent, short-term, and long-term).

## Acknowledgements

This study was part of a PhD thesis supported by Iran University of Medical Sciences, Tehran, Iran (grant No: IUMS/SHMIS-2013/342). The authors thank the directors of technical offices and their senior technicians in Iran’s Ministry of Health and medical education (MoHME), Tehran, Iran who volunteered their time to participate in this study.

## Ethical issues

The Ethics Committee of Iran University of Medical Sciences, Tehran, Iran approved the study (IUMS: 93/105/352).

## Competing interests

Authors declare that they have no competing interests.

## Authors’ contributions

Design and methodology: RZ, MH-IN, HS, and BY; Discussion and policy implications: MH-IN and HS. All authors revised, read, and approved the final manuscript.

## Authors’ Affiliations

^1^Social Determinants of Health Research Center, Lorestan University of Medical Sciences, Khorramabad, Iran. ^2^Department of Health Services Management, School of Medical Management and Information Sciences, Iran University of Medical Sciences, Tehran, Iran. ^3^Knowledge Utilization Research Center, Tehran University of Medical Sciences, Tehran, Iran. ^4^Department of Epidemiology & Biostatistics, School of Public Health, Tehran University of Medical Sciences, Tehran, Iran.

## Key messages

**Implications for policy makers**

- SUPPORT tools can be practical guidelines for health policy-making in developing countries.
- There are opportunities for all policy-makers to improve the process of evidence utilization for policy-making on the basis of SUPPORT tools.

**Implications for public**

Health policies adopted on the basis of valid research evidence are likely to meet the health goals. Evidence shows that health systems often neglect the appropriate use of research evidence which can lead to inefficiency and inequality. Improving the use of research evidence on the part of health policy-makers will probably lead to higher health benefits and better use of resources.
